# The effects of positive or negative self-talk on the alteration of brain functional connectivity by performing cognitive tasks

**DOI:** 10.1038/s41598-021-94328-9

**Published:** 2021-07-21

**Authors:** Junhyung Kim, Joon Hee Kwon, Joohan Kim, Eun Joo Kim, Hesun Erin Kim, Sunghyon Kyeong, Jae-Jin Kim

**Affiliations:** 1 Institute of Behavioral Science in Medicine, Yonsei University College of Medicine, 50-1 Yonsei-ro, Seodaemun-gu, Seoul, 03722 Republic of Korea; 2 Department of Psychiatry, Korea University Guro Hospital, Korea University College of Medicine, Seoul, Republic of Korea; 3 Department of Psychiatry, Gangnam Severance Hospital, Yonsei University College of Medicine, Seoul, Republic of Korea; 4 Department of Communication, Yonsei University, Seoul, Republic of Korea; 5 Graduate School of Education, Yonsei University, Seoul, Republic of Korea

**Keywords:** Neuroscience, Psychology

## Abstract

Self-talk can improve cognitive performance, but the underlying mechanism of such improvement has not been investigated. This study aimed to elucidate the effects of self-talks on functional connectivity associated with cognitive performance. We used the short form of Progressive Matrices Test (sRPM) to measure differences in performance improvements between self-respect and self-criticism. Participants were scanned using functional magnetic resonance imaging in the following order: baseline, during-sRPM1, post-sRPM1, self-respect or self-criticism, during-sRPM2, and post-sRPM2. Analysis was conducted to identify the self-talks' modulatory effects on the reward-motivation, default mode, and central-executive networks. Increase in sRPM2 score compared to sRPM1 score was observed only after self-criticism. The self-talk-by-repetition interaction effect was not found for during-sRPM, but found for post-sRPM; decreased nucleus accumbens-based connectivity was shown after self-criticism compared with self-respect. However, the significant correlations between the connectivity change and performance change appeared only in the self-respect group. Our findings showed that positive self-talk and negative self-talk differently modulate brain states concerning cognitive performance. Self-respect may have both positive and negative effects due to enhanced executive functions and inaccurate confidence, respectively, whereas self-criticism may positively affect cognitive performance by inducing a less confident state that increases internal motivation and attention.

## Introduction

Self-talk is the systematic use of cue words in a silent or vocalized dialog with one's self. This process has two conceptual properties: the form of verbalizations is an essential requirement and the sender of the message is also the receiver^1^. Since self-talk has beneficial effects on attention^2^ and emotion regulation^3^, it is widely used for performance enhancement in sports^1,4^, academic engagement^5^, and regulating anxiety or depression in a clinic^6,7^. Self-talk with positive contents can help with promoting positive psychological states and regulating cognitions^8,9^, whereas self-talk with negative contents is associated with emotional ill-being^10^. However, some studies have presented that negative self-talk can improve physical performance^11,12^. How negative self-talk can be beneficial in performance improvement has been explained by several hypotheses, such as motivational interpretation^13^, reverse reflection of confidence^14^, stimulating efforts to avoid a negative outcome^15^, and viewing negative self-talk as a challenge^12^.

Our research group previously reported the modulation effects of positive and negative self-talks on brain connectivity as measured by functional magnetic resonance imaging (fMRI). For example, posterior cingulate cortex (PCC)-based and ventromedial prefrontal cortex (VMPFC)-based functional connectivity for investigating the default mode network (DMN) showed that gratitude interventions modulated connectivity among motivation-related regions, including the nucleus accumbens (NA), whereas resentment interventions made considerable alteration in the connection with the DMN and task-positive regions^16^. Self-respect altered only the PCC-frontoparietal connection, whereas self-criticism changed the wide range of the self-referential, default mode, and reward-motivation networks^17^. Through these studies, the modulation effects of self-talk on brain connectivity have been revealed, but the brain basis of performance improvement due to positive and negative self-talks remains uncertain.

One of the variables measuring performance improvement is fluid intelligence, which is minimally dependent on language and acquired knowledge^18^. The Raven's Progressive Matrices (RPM) test is one of tools for measuring it^19^. Bilateral frontal (i.e., dorsolateral prefrontal cortex, DLPFC) and parietal (i.e., intraparietal sulcus, IPS) regions have been associated with fluid cognitive processes induced by the RPM task^20,21^. Performance of fluid intelligence tests can be affected by psychological states, such as depression^22^ or psychosis^23^. Another study reported performance improvement in anagram-solving tasks related to fluid intelligence after interrogative self-talk^24^. Taken together, it is worth studying the effects of self-talk on brain networks during psychological states in terms of changes in fluid intelligence-related performance, but this has not been demonstrated yet to our knowledge.

The effects of task-related cognitive load on functional connectivity have provided useful insights not only into ongoing processes concerning cognitive functions, but also subsequent processes during post-task resting-state. For example, studies investigating post-task resting-state have shown that changes in connectivity reflect recent visual/cognitive experience^25^ and further predict subsequent cognitive performance^26,27^. Additionally, post-task resting-state connectivity is associated with experience-induced plasticity^28^. Other examples include post-task changes related to cognitive functions, such as episodic memory^29^ and visual perception^30^. Taken together, when investigating the effects of self-talk on cognitive performance, it would be meaningful to evaluate both during-task and post-task changes.

The present study aimed to elucidate the effects of positive and negative self-talks on alterations in functional connectivity related to performance of fluid intelligence tests. For this aim, seed-based connectivity was investigated on fMRI data, which were obtained while and after performing the RPM tasks before and after the self-respect or self-criticism task. Given the two conceptual properties of self-talk, the verbalized form and the identity of the sender and receiver of the message, and the experimental requirement that all participants be given the same conditions, the self-respect and self-criticism tasks consisted of having the participants read and record the sentences expressing themselves with “I” as the subject in advance, and having them repeat the contents while listening to the recordings in the fMRI experiment. Our hypotheses were that both the self-respect and self-criticism tasks would induce performance improvement in the RPM tasks, whereas the during-task and post-task modulations of functional connectivity underlying these improvements would be different between the two self-talk tasks in the reward-motivation network, DMN, and task-positive network in the brain. Based on these hypotheses, the seeds were defined as the NA and VMPFC in the reward-motivation network, the PCC in the DMN, and the DLPFC and IPS in the task-positive network.

## Results

### Participants’ psychological scale scores and task performances

The two cognitive tasks during fMRI scanning were short forms of the RPM test, which were referred to as sRPM1 and sRPM2. Although a total of 46 participants were scanned, data from those with the sRPM1 score of seven (two standard deviations lower than mean) or less were excluded from the analysis because exceptionally low scores of the first cognitive task suggesting poor attention might have an excessive and inappropriate impact on the analysis. Three participants from the self-criticism group met this criterion and were excluded. The final analysis was conducted on the self-respect group of 23 participants and the self-criticism group of 20 participants, and there was no significant group difference in age (22.48 ± 2.13 years old and 23.90 ± 2.65 years old, respectively) and sex (12 males and 13 males, respectively).

Psychological scale scores and sRPM scores are presented in Table 1. The Rosenberg Self-Esteem Scale (RSES) score, Hospital Anxiety and Depression Scale (HADS)—anxiety score, and HADS—depression score did not significantly differ between the two groups. Compared to sRPM1 score, a significant increase in sRPM2 score was observed in the self-criticism group (*t*_19_ = 2.80, *p* = 0.011, *Cohen’s d* = 0.63), but not in the self-respect group (*t*_*22*_ = 1.29*, p* = 0.212*, Cohen’s d* = *0.27*). Accordingly, the self-criticism group showed significantly higher sRPM increase rate than the self-respect group (*F*_1,40_ = 5.08, *p* = 0.030, η^2^ = 0.113).

**Table 1:** Summary of psychological assessments and task performances in each self-talk group.

Variable	Self-respect group (n = 23)	Self-criticism group (n = 20)	*t*	*p*
RSES	30.65 ± 7.21	31.10 ± 5.25	0.23	0.819
**HADS**				
Anxiety score	4.83 ± 2.99	4.80 ± 2.71	− 0.03	0.976
Depression score	5.13 ± 3.36	5.25 ± 2.69	0.13	0.899
**sRPM performances**				
sRPM1 score	12.74 ± 1.79	12.50 ± 2.26	− 0.39	0.701
sRPM2 score	13.30 ± 2.41	14.20 ± 1.70	1.50	0.141
sRPM increase rate (%)	5.61 ± 18.77	17.07 ± 24.77	5.08	0.030

Values are means ± standard deviation.

*RSES* Rosenberg self-esteem scale, *HADS* hospital anxiety and depression scale, *sRPM* short form of Raven’s Progressive Matrices. sRPM increase rate = [(sRPM2 score – sRPM1 score)/sRPM1 score] × 100.

### Changes in during-sRPM state functional connectivity

Results of the seed-based connectivity analysis for states during the sRPM tasks are presented in Table 2. The main effect of self-talk was found only in DLPFC-based connectivity with the right precentral gyrus (PrCG), in which the connectivity strengths were significantly higher in the self-respect group than in the self-criticism group (*t*_41_ = 5.72, *p* < 0.001, *Cohen’s d* = 0.89). The main effect of repetition was seen in the connections of NA—right lateral occipital cortex (LOC), VMPFC—bilateral parietal operculum cortex (POC), and PCC—left PrCG, in which the connectivity strengths were all significantly increased during sRPM2 compared with sRPM1 (*t*_41_ = 6.10, *p* < 0.001, *Cohen’s d* = 0.95; *t*_41_ = 6.00, *p* < 0.001, *Cohen’s d* = 0.93; *t*_41_ = 5.23, *p* < 0.001, *Cohen’s d* = 0.80; and *t*_41_ = 7.11, *p* < 0.001, *Cohen’s d* = 1.11, respectively), and in the connection of DLPFC—right middle temporal gyrus (MTG), in which the connectivity strengths were significantly decreased during sRPM2 compared with sRPM1 (*t*_41_ = − 6.40, *p* < 0.001, *Cohen’s d* = 0.99). Meanwhile, there was no inter-regional connectivity showing the self-talk × repetition interaction effect.

**Table 2:** Results of the seed-based functional connectivity analysis for brain states while performing the short form of Raven’s Progressive Matrices (sRPM) in the two different self-talk groups: self-respect and self-criticism.

Source	Target	MNI coordinate			N_vox_	Z_max_	Post-hoc analysis
		x	y	z			
**Main effect of self-talk (self-respect versus self-criticism)**							
NA/VMPFC/PCC	–						
DLPFC	R. precentral gyrus	36	− 26	54	298	5.14	SR > SC
IPS	–						
**Main effect of repetition (during-sRPM1 versus during-sRPM2)**							
NA	R. lateral occipital cortex	42	− 78	− 22	209	5.68	sRPM1 < sRPM2
VMPFC	L. parietal operculum cortex	− 54	− 26	20	278	6.46	sRPM1 < sRPM2
	R. parietal operculum cortex	54	− 28	26	119	4.24	sRPM1 < sRPM2
PCC	L. precentral gyrus	− 56	04	16	452	7.36	sRPM1 < sRPM2
DLPFC	R. middle temporal gyrus	60	− 32	− 14	230	− 7.05	sRPM1 > sRPM2
IPS	–						
**Interaction effect: self-talk × repetition**							
NA/VMPFC/PCC/DLPFC/IPS	–						

*MNI* Montreal neurological institute, *N*_*vox*_ numbers of voxels, *Z*_*max*_ maximum z-value within the cluster, *L.* left, *R.* right, *NA* nucleus accumbens, *VMPFC* ventromedial prefrontal cortex, *PCC* posterior cingulate cortex, *DLPFC* dorsolateral prefrontal cortex, *IPS* Intraparietal sulcus.

### Changes in post-sRPM resting-state functional connectivity

**Figure 1:**
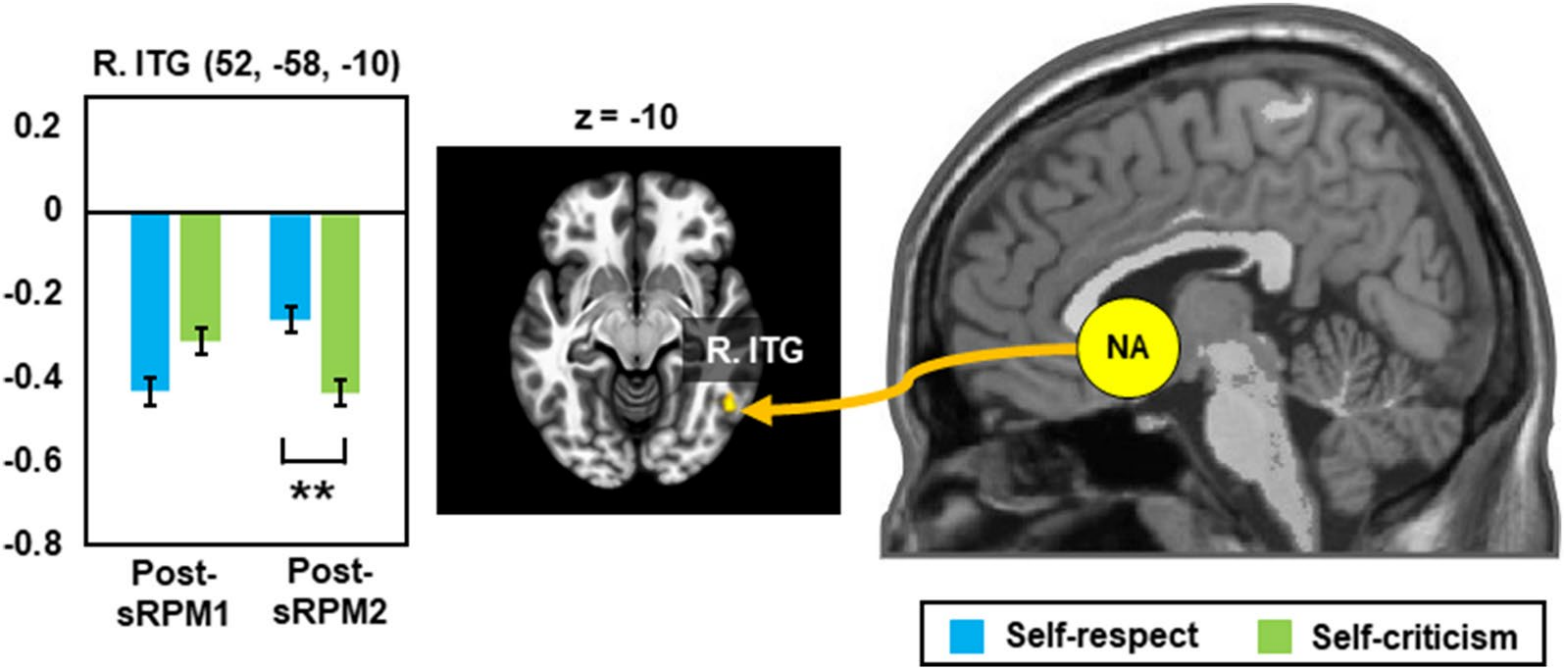
Post-hoc analysis of repeated measure analysis of covariance for resting-state functional connectivity after performing the short forms of Raven’s Progressive Matrices (sRPM1 and sRPM2). *R.* right, *NA* nucleus accumbens, *ITG* inferior temporal gyrus. *p < 0.05, **p < 0.01 for post-hoc comparisons after Bonferroni correction.

Results of the seed-based functional connectivity analysis for resting-states after the sRPM tasks are presented in Table 3. The main effect of self-talk was found only in NA-based connectivity with the left MTG and right LOC. Post-hoc tests showed that the connectivity strengths of these two were significantly higher in the self-respect group than in the self-criticism group (*t*_41_ = 5.20, *p* < 0.001, *Cohen’s d* = 0.81; and *t*_41_ = 5.25, *p* < 0.001, *Cohen’s d* = 0.82, respectively). Although no repetition effect was seen, the self-talk × repetition interaction effect was observed in NA-based connectivity with the right inferior temporal gyrus (ITG). As shown in Fig. 1, post-hoc tests showed that the NA-right ITG connectivity strengths in the post-sRPM1 resting-state did not differ between the two groups, whereas those in the post-sRPM2 resting-state were significantly higher in the self-respect group than in the self-criticism group (*t*_41_ = 5.27, p = 0.001, *Cohen’s d* = 0.82).

**Table 3:** Results of the seed-based functional connectivity analysis for resting-states after performing the short form of Raven’s Progressive Matrices (sRPM) in the two different self-talk groups: self-respect and self-criticism.

Source	Target	MNI coordinate			N_vox_	Z_max_	Post-hoc analysis
		x	y	z			
**Main effect of self-talk (self-respect versus self-criticism)**							
NA	L. middle temporal gyrus	− 66	− 48	04	144	5.71	SR > SC
	R. lateral occipital cortex	54	− 68	12	142	5.25	SR > SC
VMPFC/PCC/DLPFC/IPS	–						
**Main effect of repetition (post-sRPM1 versus post-sRPM2)**							
NA/VMPFC/PCC/DLPFC/IPS	–						
**Interaction effect: self-talk × repetition**							
NA	R. inferior temporal gyrus	52	− 58	− 10	113	5.04	See Fig. 1
VMPFC/PCC/DLPFC/IPS	–						

*MNI* Montreal neurological institute, *Nvox* numbers of voxels, *Z* maximum z-value within the cluster, *L.* left, *R.* right, *NA* nucleus accumbens, *VMPFC* ventromedial prefrontal cortex, *PCC* posterior cingulate cortex, *DLPFC* dorsolateral prefrontal cortex, *IPS* Intraparietal sulcus, *SR* self-respect, *SC* self-criticism.

### Changes in functional connectivity and increase of sRPM scores

**Figure 2:**
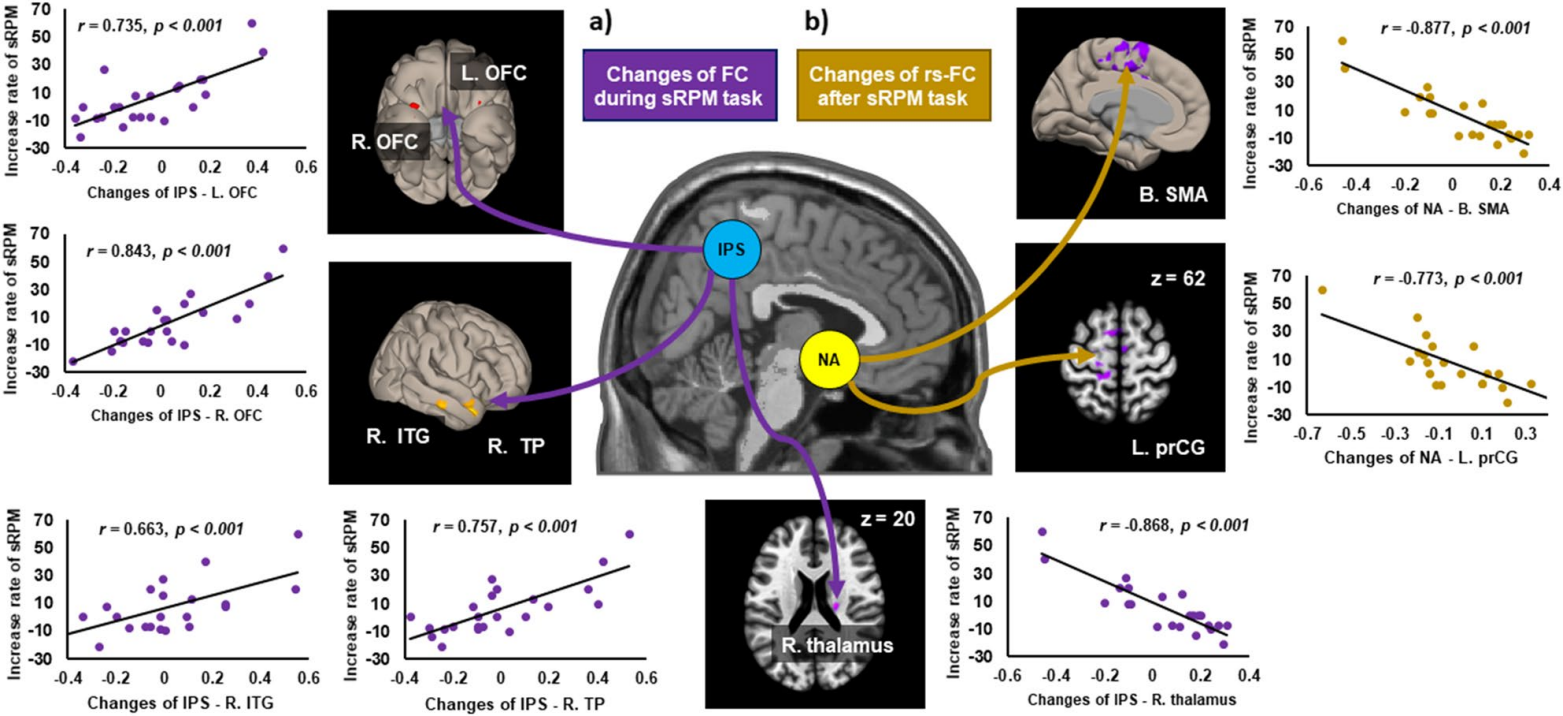
Scatter plots showing the relationships between during-task (**a**) and post-task (**b**) changes in inter-regional functional connectivity (FC) and score increase rates of the short form of Raven’s Progressive Matrices (sRPM) in the self-respect group. rs-FC, resting-state functional connectivity; *L.* left, *R.* right, *B.* bilateral, *NA* nucleus accumbens, *IPS* intraparietal sulcus, *SMA* supplementary motor area, *PrCG* precentral gyrus, *OFC* orbitofrontal cortex, *ITG* inferior temporal gyrus, *TP* temporal pole.

Table 4 presents brain regions that showed the significant association between changes in inter-regional functional connectivity and sRPM increase rates. In the self-respect group, significant correlations between changes in during-sRPM state connectivity and sRPM increase rates were observed in IPS-based connectivity with the bilateral orbitofrontal cortex (OFC) (positive correlation), right temporal pole (positive correlation), right ITG, and right thalamus (negative correlation) (Fig. 2a). The self-respect group also showed negative correlations between changes in post-sRPM resting-state connectivity and sRPM increase rates in NA-based connectivity with the left supplementary motor area (SMA) and left PrCG (Fig. 2b). However, the self-criticism group showed no significant correlations for both during-sRPM state and post-sRPM resting-state connectivity.

**Table 4:** Significant relationships between score increase rate of the short form of Raven’s Progressive Matrices (sRPM) and changes of functional connectivity in during-sRPM states and in post-sRPM resting-states in each of the self-respect and self-criticism groups.

Group	Seed	Target	MNI coordinate, mm			N_vox_	Z_max_
			x	y	z		
**Changes in functional connectivity between during-sRPM1 and during-sRPM2**							
Self-respect	NA/VMPFC/PCC/DLPFC	–					
	IPS	R. orbitofrontal cortex	30	20	− 28	247	7.96
		L. orbitofrontal cortex	− 32	24	− 24	76	5.72
		R. temporal pole	48	04	− 30	116	7.59
		R. inferior temporal gyrus	62	− 22	− 20	61	5.41
		R. thalamus	20	− 18	20	64	− 7.34
Self-criticism	NA/VMPFC/PCC/DLPFC/IPS	–					
**Changes in functional connectivity between post-sRPM1 and post-sRPM2**							
Self-respect	NA	B. supplementary motor area	− 12	− 36	60	1236	− 8.07
		L. precentral gyrus	− 20	− 22	62	93	− 5.26
Self-criticism	VMPFC/PCC/DLPFC/IPS	–					
	NA/VMPFC/PCC/DLPFC/IPS	–					

*MNI* Montreal neurological institute, *N*_*vox*_ numbers of voxels, *Z*_*max*_ maximum z-value within the cluster, *L.* left, *R.* right, *B.* bilateral, *NA* nucleus accumbens, *VMPFC* ventromedial prefrontal cortex, *PCC* posterior cingulate cortex, *DLPFC* dorsolateral prefrontal cortex, *IPS* intraparietal sulcus.

## Discussion

To identify the difference in the effects of positive and negative self-talks on functional connectivity concerning alterations in cognitive performance, we investigated changes in during-sRPM and post-sRPM connectivity before and after self-respect versus self-criticism. Behavior data showed that sRPM increase was significantly higher after self-criticism than after self-respect, suggesting that negative self-talk may be more beneficial in the improvement of cognitive performance than positive self-talk. The modulation effects on various networks and associations between connectivity alterations and performance changes also differed between the self-talk groups, suggesting that the modification of brain connectivity may play a mediating role in the effects of self-talks on the promotion of cognitive performance.

### Self-talks and task repetition

The repetition effect was shown for during-sRPM states, but not for post-sRPM resting-states. DLPFC-MTG connectivity decreased in during-sRPM2 compared with during-sRPM1. The DLPFC-based network is necessary for key competencies of intelligence and executive functions^31^, and MTG activity may involve increases of task demand^32^. Task repetition within a short time, similar to the current study, degrades performance due to cognitive fatigue^33,34^. Cognitive fatigue can be motivational fatigue related to a system that maintains motivation through monitoring internal states^35^, induce decreases of attention-related network connectivity^36^, and reduce the demand for cognitive tasks of the same difficulty level^34^. Therefore, decreased DLPFC-MTG connectivity may reflect a decline of cognitive demand associated with cognitive fatigue.

NA-, PCC-, VMPFC-based connectivity increased in during-sRPM2 compared with during-sRPM1. The NA-related network is engaged in reward prediction associated with motivation^37,38^. The NA involves not only external reward but also novelty of stimulus^39^, and thus task repetition can induce deactivation of the reward system. Cognitive fatigue negatively affects motivation^35^, whereas self-talks provided as self-related information positively affect individual performance concerning motivation^1^. Therefore, the change in NA-based connectivity seems to support motivational interpretation of self-talks. Alternatively, it may reflect inaccurate confidence associated with familiarity according to task repetition. Inaccurate confidence means high confidence that does not match actual accuracy^40^. Increased NA-based connectivity was observed in the right LOC. Magnetic stimulation of the occipital cortex reduces task accuracy and, conversely, increases confidence^41^. The increase in confidence or lack of attention increases the variability of the internal signal for stimuli, thereby inducing inaccurate selection^42^.

Given that the PCC and VMPFC are nodes of the self-referential network and DMN^43^, increased PCC-PrCG or VMPFC-POC connectivity is likely to be induced by self-talks rather than task repetition. There is a recent report that connectivity of the self-referential network and DMN negatively correlated with cognitive fatigue^34^. Connectivity between the DMN and PrCG is related to associative learning or motivational assignments to the ongoing motor task demands^44^. POC activity involves retrieving recently learned information^45^. Therefore, the self-referential network and DMN activated by self-talks may provide an environment that can lead to performance improvement despite cognitive fatigue.

### Effects of self-respect

The main effect of self-talk was observed in DLPFC-PrCG connectivity for during-sRPM states and in NA-MTG and NA-LOC connectivity for post-sRPM resting-states. Self-respect lead to stronger connectivity in all of these connections than self-criticism. Given a key role of the DLPFC in executive functions^31^ and responsibility of the PrCG for implementing corrective strategies^46^, robust DLPFC-PrCG connectivity suggests that self-respect may be more beneficial for executive functions than self-criticism. The results of NA-based connectivity are almost unheard of, making it difficult to interpret their meaning. MTG activity is influenced by subjective confidence in one’s accuracy of tasks^47,48^, and LOC activity is associated with an event-sequence that affects the reward system^49^. Confidence is an environmental factor in the computational model of motivation^50,51^. Therefore, NA-based connectivity for post-sRPM resting-states suggests that individuals who have experienced self-respect may be more confident than those who experienced self-criticism. Alternatively, these results may reflect the inference that motivational interpretation of positive self-talk may be related to an induced environmental factor, such as more enhanced confidence. Considering that there is an association between external stimulus on the occipital cortex and increased inaccurate confidence^41^, robust NA-LOC connectivity in the self-respect group may involve inaccurate confidence that can negatively affect cognitive performance with impulsiveness.

Only the self-respect group showed significant correlations between connectivity changes and performance changes for both during-sRPM and post-sRPM states. However, since the sRPM scores were not changed in this group, the connectivity changes were not large enough to appear as a behavioral change. Alternatively, it can be because self-respect has both positive and negative effects on cognitive performance. Specifically, changes in IPS-OFC connectivity during s-RPM tasks positively correlated with sRPM increase. The parietal network plays an essential role in cognitive reasoning^33^ and is modulated by psychological interventions^52^. The OFC is engaged in coordination and synthesis of visual and motor representations and in performance on processing speed^53^. Therefore, our result may be associated with altered brain states that are beneficial for potential performance improvement induced by self-respect. In contrast, sRPM increase negatively correlated with changes in NA-based connectivity after cognitive tasks, suggesting that increased NA-based connectivity by self-respect may negatively affect cognitive performance. About NA-based connectivity associated with confidence, self-respect may adversely affect cognitive performance by increasing impulsiveness, similar to risk behaviors in association with inaccurate confidence dissociated from actual results^54^. Taken together, the effects of self-respect on cognitive performance seem both negative, due to impulsivity related to inaccurate confidence, and positive, due to performance improvement related to enhanced executive functions. Since there are various methods other than self-respect for positive self-talk, additional studies using other self-talk tasks are needed to understand its effects on cognitive performance.

### Effects of self-criticism

Compared to sRPM1 score, sRPM2 score was significantly increased in the self-criticism group, but not in the self-respect group, and thus sRPM increase rate was significantly higher in the self-criticism group than in the self-respect group. Increased sRPM score in the self-criticism group is consistent with previous findings for the beneficial effect of negative self-talk on enhancing performance^12,55^. This effect may be because negative self-talk has a significant influence on attention. Negative stimuli increase attention to a subsequent stimulus compared with positive stimuli^56^. Alternatively, given that motivation is a critical factor in maintaining attention^57^, self-criticism may reduce cognitive fatigue-related inattention by being interpreted more motivational. This motivational interpretation may be either because individuals try to avoid negative results on their own through negative self-talk^15^ or accept it as a challenge^12^. Despite performance improvement after self-criticism, it was not correlated with connectivity change, maybe due to the ceiling effect as most participants showed an increase in performance.

This behavioral result is supported by the self-talk effects on DLPFC-PrCG connectivity for during-sRPM states and NA-MTG and NA-LOC connectivity for post-sRPM resting-states, which should be considered contrary to self-respect. In particular, considering that less confident state can induce motivation^58^, these findings suggest that confidence lowered by self-criticism and subsequent motivational interpretation can lead to performance improvement. This is also supported by NA-ITG connectivity in post-sRPM resting-states, which showed no group difference before self-talk, but was decreased after self-criticism. Since enhanced ITG activity is involved in more confident states^40,59^ and the less robust ITG activity is associated with the greater internal motivation^60^, decreased NA-ITG connectivity can reflect decreased confidence and increased motivation induced by self-criticism.

Although self-criticism was better at increasing sRPM scores than self-respect, it cannot be generalized that negative self-talk will have a superior effect on performance improvement than positive self-talk. The effect of self-talk decreases as repeated over time^61^, and long-term exposure to negative self-talk has harmful effects^1^. Therefore, our findings on the effects of negative self-talk should be interpreted only from a short-term perspective. Further studies are needed on the long-term effects of negative self-talk on changes in brain connectivity that underlie cognitive performance changes.

## Limitations

There are some limitations to our study, which can constrain the interpretations. First, our study samples consisted of young, healthy, college students who were likely of higher intellectual capacity than average. Thus, it is uncertain whether the results will be similar in the general population. Second, sRPM scores represented mainly fluid intelligence, not overall cognitive performance, and the type and difficulty of cognitive tasks were not considered. Third, as the current study compared two groups divided according to the type of self-talk tasks, there is a possibility that confounding factors may be involved. In fact, it might be desirable to see the effect of performing both self-respect and self-criticism in a single group. To do this, however, the experimental time given to one participant would be too long, and the sRPM sets would have to be doubled. These could lead to other confounding factors, and thus we had no choice but to choose the current two-group design. Fourth, the current study design did not include non-self-reflective neutral control task, and thus analysis for common effects of repetitions was inevitably lacking. Finally, the current study did not monitor physiological data, including heart rate, which can affect cognitive performance.

## Conclusions

The current study is the first study that directly compared the effects of positive and negative self-talks concerning both cognitive performance and functional connectivity. By identifying brain responses to self-talks, our study presented that both types of self-talks can enhance cognitive performance through different brain changes related to motivation. In summary, the effects of self-respect on cognitive performance seem both negative, due to impulsivity related to inaccurate confidence, and positive, due to performance improvement related to enhanced executive functions. On the other hand, self-criticism may induce an increase in cognitive performance, maybe due to a less confident state that elevates internal motivation and attention. Additional studies are needed to elucidate the modulation of confidence and motivation concerning both self-talk and cognitive performance. Moreover, further studies need to address the long-term effect of positive and negative self-talks on changes in brain connectivity that underlie cognitive performance changes.

## Materials and methods

### Participants

Participants were 46 healthy college student volunteers (23.17 ± 2.39 years old, 25 males and 21 females) with no past or present history of major neurological or psychiatric disorders and medical diseases that can cause dysfunctions in cognitive performance and no experience of any form of psychological interventions including self-talks. All of them were right-handed as assessed with the Annett Handedness Inventory^62^. This study was approved by the Institutional Review Board of Yonsei University Severance Hospital and carried out in accordance with the Declaration of Helsinki. All participants voluntarily signed written informed consent, and received the same amount of Korean money 50,000 won in exchange for participation in the experiment.

### Psychological assessments

Before conducting fMRI experiments, all participants completed two self-report questionnaires. The first was the RSES, comprised of 10 items and four-point Likert scale for measuring an individual’s self-esteem^63^. The second was the HADS, comprised of 14 items (seven for anxiety and seven for depression) and four-point Likert scale for measuring an individual’s level of depression and anxiety^64^.

### Audiovisual stimuli and assignment of participants

Based on our previous study, which presented different patterns between alterations in brain connectivity by self-respect and self-criticism^17^, we prepared scripts for the two types of self-talks, which were intended to facilitate participants to focus on the feeling of self-respect or self-criticism by telling themselves in their minds how much they respect or criticize themselves. Full scripts of the text are provided in Supplementary Material S1. A 5-min audiovisual stimulus for the self-talk task was produced 1 week before the fMRI scan. We made an audio stimulus of participants’ own voice by recording their script reading and then combined it with a visual stimulus, in which the scripts were visually presented in black letters on a gray background. Assigning participants to the self-respect or the self-criticism group was done through simple randomization using a computerized random number generator.

Meanwhile, we also prepared two sets of 5-min cognitive tasks (sRPM1 and sRPM2) produced by selecting 20 questions differently out of the 60 questions of the RPM test^19^ and reducing the answer options to four. In these tasks, visual stimuli were presented on the screen for 15 s with a question placed in the center and the answer options placed in the bottom. The difficulty levels of the two tasks were set to be as similar as possible, and in a preliminary study of 10 participants other than those who participated in this fMRI experiment, the average score of sRPM1 and sRPM2 showed no statistical difference (13.50 ± 1.58 and 13.50 ± 1.72, respectively; *t*_9_ = 0.00, *p* = 1.00).

### Experimental procedure and behavioral analyses

**Figure 3:**
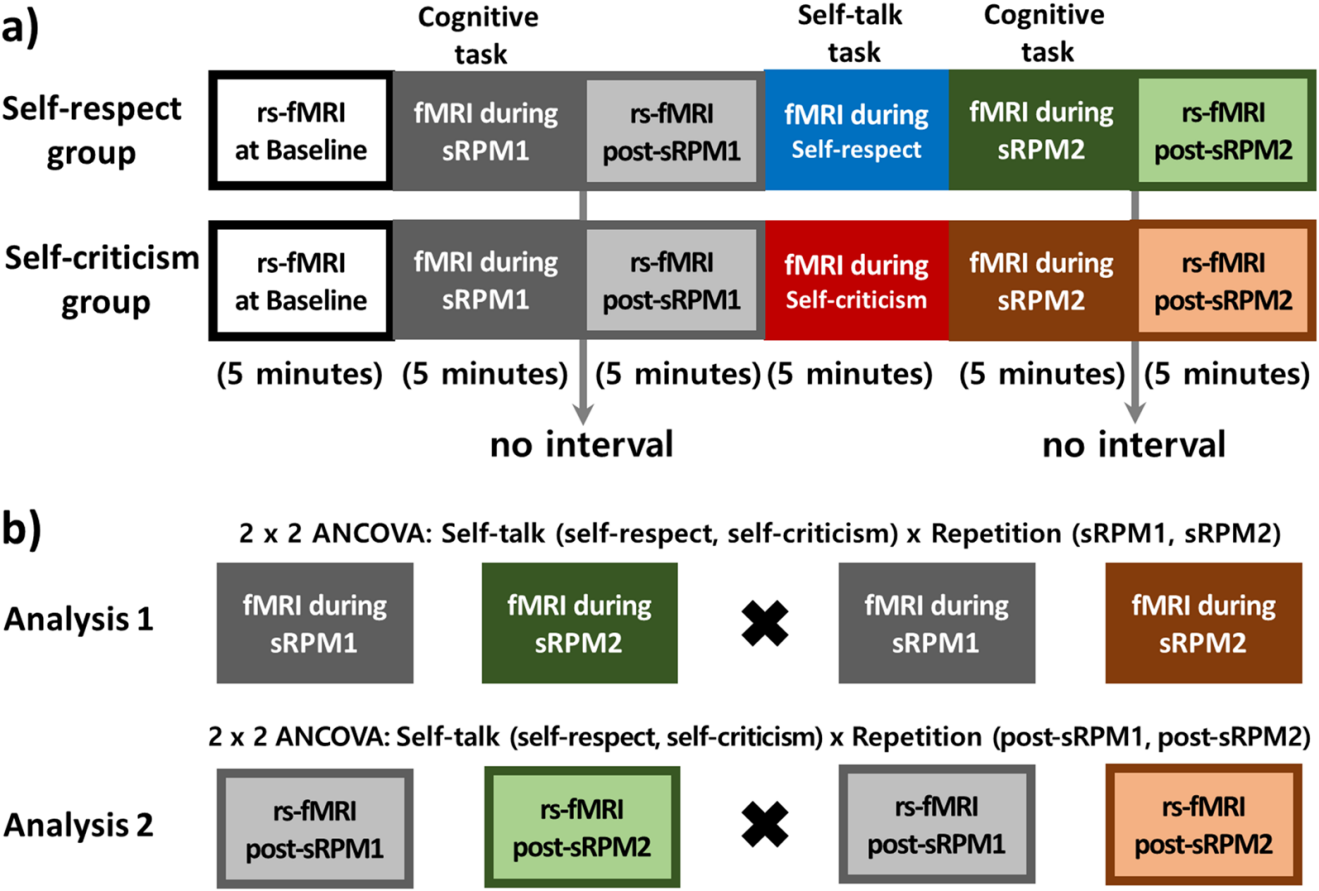
Experimental procedures of resting-state functional magnetic resonance imaging (rs-fMRI) and fMRI during the two short forms of Raven’s Progressive Matrices (sRPM 1 and sRPM2) in the self-respect and self-criticism groups (**a**), and the diagrams for statistical analysis (**b**). The order of sRPM1 and sRPM2 was counterbalanced across participants in each group. Analysis of covariance (ANCOVA) was performed for self-talk (self-respect vs. self-criticism) × repetition (during-sRPM states in Analysis 1 and post-sRPM resting-states in Analysis 2).

As shown in Fig. 3a, the experimental procedure consisted of six 5-min sessions of the fMRI scanning in the following order: baseline resting-state, first sRPM task (sRPM1), second resting-state (post-sRPM1), self-talk task, second sRPM task (sRPM2), and third resting-state (post-sRPM2). In the self-talk task sessions, the participants were instructed to focus on mental images of self-respect or self-criticism, respectfully, according to their assigned groups. Throughout these tasks, the audiovisual guidance instructed the participants to focus on the narrated scripts once, and then recite them silently, sentence by sentence, in their mind. In the session of sRPM1 or sRPM2, the participants chose their answers by pressing one of the four buttons with their index and middle fingers of both hands. The order of these two cognitive tasks was counterbalanced among the participants. During the three resting-state sessions, the participants were instructed to stare at a fixation cross presented on the screen, relax, and think of nothing in particular. For enhancing the effectiveness of the tasks, a minute-long audiovisual guides instructing how to breathe and relax or how to solve the problem were provided during a short break prior to the self-talk task and the cognitive tasks, respectively. There was no break for instruction between the sRPM task and following resting-state.

Responses for the sRPM tasks were reported as the total number of correct answers, named the sRPM1 and sRPM2 scores. For comparing the modulation effect of two self-talk tasks, we calculated the sRPM increase rate with the formula of [(sRPM2 scores − RPM1 score)/sRPM1 score] × 100 (%). We performed paired *t* tests for the sRPM1 and sRPM2 scores in each group. In addition, the effect of two self-talk tasks on the sRPM increase rate was compared using analysis of covariance (ANCOVA) with controlling for age, which is a factor related to the decline of fluid intelligence in adults^18^.

### Imaging data acquisition and preprocessing

Images were acquired using a 3.0 T MR scanner (Ingenia CX, Philips, Best, the Netherlands) with a 32-channel dS head coil. For each participant, we acquired fMRI scans using the multiband SENSitivity Encoding (SENSE) sequence (matrix size, 96 × 93; field of view, 216 mm; number of slices, 60; slice order, bottom-up and interleaved; slice thickness, 2.4 mm; echo time, 30 ms; repetition time, 800 ms; flip angle, 52°; MB factor, 6; and SENSE factor, 1). We acquired additional fMRI scans of the same parameters with two opposite phases encoding directions (anterior to posterior and posterior to anterior) to correct the geometric distortion of the multi-band fMRI data. Anatomical images were obtained in the coronal direction using a 3D T1-weighted fast gradient echo sequence (matrix size, 224 × 224; field of view, 224 mm; number of slices, 220; slice thickness, 1 mm; echo time, 4.6 ms; repetition time, 9.9 ms; and flip angle, 8°).

All fMRI scans were corrected for susceptibility-induced geometric distortions using the FSL TOPUP tool^65,66^, and the first 10 scans were discarded for magnetic field stabilization. Preprocessing of the functional data was carried out in Montreal Neurological Institute (MNI)-space using CONN functional connectivity toolbox (ver.19.c, http://www.nitrc.org/projects/conn) and Statistical Parametric Mapping 12 (SPM12, http://www.fil.ion.ucl.ac.uk/spm). The remaining 375 functional scans for each run were realigned to the first scan, and temporal misalignment between different slices was corrected using the slice-timing correction procedure. Considering the pervasive impact of head motion on measures of functional connectivity^67–69^, correcting for motion by regressing out both motion parameters and specific frames with motion outliers was performed using the Artifact Rejection Toolbox (ART; http://www.nitrc.org/projects/artifact_detect/) implemented in CONN for outlier detection and scrubbing to create confound regressors for motion parameters (global-signal Z value = 9; subject motion = 2 mm). Functional and structural data were normalized into standard MNI space and segmented into tissue classes of grey matter, white matter, and cerebrospinal fluid using SPM12 unified segmentation and normalization procedure^70^. To increase signal-to-noise ratio and reduce the influence of variability in functional data and gyral anatomy across subjects, functional smoothing was conducted using spatial convolution with a Gaussian kernel of 6 mm full width at half maximum. Functional data were then temporally band-pass filtered (0.009–0.08 Hz) to remove low-frequency drift while minimizing the influence of physiological, head-motion, and other noise sources^71^.

### Seed-based functional connectivity

Based on our hypothesis that the NA and VMPFC in the reward-motivation network, the PCC in the DMN, and the DLPFC and IPS in the task-positive network would involve changes in the performance of cognitive tasks depending on the two contrasting self-talk tasks, a seed-based whole-brain approach was conducted using these five as the regions of interest (ROIs). Their MNI coordinates (x/y/z) were determined by referring to the results of previous studies: the NA, ± 12/8/− 8^72^, the VMPFC, 9/51/16 and PCC, 1/− 26/31^73^, and the DLPFC, ± 42/24/24 and IPS, ± 36/− 54/39^74^. The ROIs were defined as a sphere of 3-mm radius around the selected MNI coordinates.

In the first-level analysis, the levels of functional connectivity between each ROI and every voxel in the brain were computed as the Fisher-transformed bivariate correlation coefficients between the time series of functional data. Potential confounding factors including cerebral white matter and cerebrospinal areas, estimated subject-motion parameters, identified outlier scans, constant and first-order linear session effects were estimated and regressed out using CONN’s default denoising pipeline implement an anatomical component-based noise correction procedure (aCompCor). We conducted two repeated-measures ANCOVA controlling for age, gender, and head motion parameters to explore any significant differences in functional connectivity related to cognitive performs changes according to two self-talk tasks (Fig. 3b). In ANCOVA model I, we considered two self-talk tasks and two states during cognitive tasks: 2 (self-talk, self-respect versus self-criticism) × 2 (repetition, sRPM1 versus sRPM2). In the ANCOVA model II, we considered two self-talk tasks and two resting-states after cognitive tasks: 2 (self-talk, self-respect versus self-criticism) × 2 (repetition, post-sRPM1 versus post-sRPM2). Direct comparison of fMRI data obtained during the self-respect and self-criticism task sessions was excluded from the analysis because this issue did not fit the purpose of the study to elucidate the effect of self-talks on cognitive performance and was addressed more intensively in our previous study^17^. Statistical inferences for identifying brain regions showing main and interaction effects were performed at a threshold of the cluster-level false-discovery-rate-corrected *p* (*p*_FDR_) < 0.05 with the cluster-forming threshold at the voxel level of uncorrected *p* < 0.001. Post-hoc two-sample *t*- or paired *t* tests were conducted to compare the mean beta values of all voxels in the significant clusters, and significant results were identified based on Bonferroni-corrected *p* < 0.05.

In addition, to identify the brain regions that demonstrated significant associations between changes before and after self-talk tasks in during-sRPM state or post-sRPM resting-state functional connectivity and changes in the performance of cognitive tasks, functional connectivity difference maps of each ROI calculated by extracting during-sRPM1 from during-sRPM2 and extracting post-sRPM1 from post-sRPM2 were applied for linear regression analysis using the sRPM increase rates as a dependent variable. Voxelwise-analyses were performed, and the significance was considered at *p*_FDR_ < 0.05 among clusters at a cluster-defining threshold of uncorrected *p* < 0.001. Next, we computed the Pearson correlation between the mean beta values of all the significant clusters in functional connectivity difference maps and the sRPM increase rates.

## Supplementary Information

Supplementary Information.
