# Supplementary material for: The effects of positive or negative self-talk on the alteration of brain functional connectivity by performing cognitive tasks
Source: Sci Rep. 2021 Jul 21;11:14873. doi: 10.1038/s41598-021-94328-9 (PMC8295361; doi:10.1038/s41598-021-94328-9)
Supplement: Supplementary file 1 — Supplementary Information. [file 41598_2021_94328_MOESM1_ESM.docx]

**<Supplementary Material S1>**

**Full scripts of the text for the self-respect and self-criticism tasks**

(The scripts were presented in Korean in the experiment, but here they are translated in English to aid understanding.)

1. Self-respect

I have many strengths. I am well aware of these strengths. I have many great qualities. I am well aware of these qualities. There are many wonderful sides to me. I am aware of my wonderful sides**.** If I think about it, I know how to do a lot of things. If I think about it, there are many things that I do well. I believe in my abilities. I have a potential. I have tremendous potentials. I know the things that I could do. And I can do those things. I can do things well. When I put my mind on something, I follow through someday. My abilities continue to grow. I feel pride in myself. I feel proud when I reflect on myself. I have overcome the difficulties well. And I can continue to overcome the difficulties well. I believe in myself. I can do it. I am proud of myself. I love myself. I truly respect myself. I respect and love myself. I love and respect the people around me. The people around me love and respect me. I love and respect my friends. My friends love and respect me. I am the owner of my life. I choose my own life. No one else but I determine my life. My life depends on my decisions. I determine the success of my life. I own my life. I am the owner of my life. I respect myself. I *truly* respect myself.

2. Self-criticism

I have made many mistakes in my life. I know what I have done wrong. It is all my fault. I have many weaknesses. I am well aware of my weaknesses. I know what I can’t do. I can hardly do anything well. I can’t do anything. There is nothing I can do properly. My life is a mess. There is nothing good about me. I look pathetic. I am embarrassed of myself. If others find out about the real me, they will be disappointed. I don’t like myself. I hate myself. I am stupid. I am an idiot. I can’t do anything. I have done nothing right. I hate myself. Everyone else will all hate me. Everyone will be disappointed in me. Everyone will not like me. I will not be loved by anyone. I am a person who does harm to other people. I make other people uncomfortable. I continue to fail. I fail because I fall short. I get nervous when something important comes up. I repeatedly make mistakes. I will continue to fail. I am ruining my life. I hate myself. I am selfish. I am greedy. I am a cheap shot. I am angry at myself. I am ashamed of myself from others. I am sorry for those I love. Something is wrong with me. I *truly* hate myself.
